# Urine-Based cfDNA Ensemble Modeling for Early Detection of Bladder Cancer Using Whole-Genome Methylation Sequencing

**DOI:** 10.3390/cancers18050767

**Published:** 2026-02-27

**Authors:** Taehoon Kim, Dongju Shin, Hyun Kyu Ahn, Young Joon Moon, Duhee Bang, Kwang Hyun Kim

**Affiliations:** 1 Department of Chemistry, Yonsei University, 50 Yonsei-ro, Seodaemun-gu, Seoul 03722, Republic of Korea; ktae5@yonsei.ac.kr (T.K.);; 2 Department of Urology, Ewha Womans University Seoul Hospital, Seoul 07804, Republic of Korea

**Keywords:** bladder cancer, urine cell-free DNA, whole-genome methylation sequencing, multi-feature ensemble model, non-invasive cancer detection

## Abstract

Early detection of bladder cancer poses a major challenge for liquid biopsy due to limited tumor burden and low abundance of tumor-derived DNA. In such low-signal settings, detection sensitivity critically depends on both biofluid selection and effective integration of weak, distributed molecular signals. We analyzed Enzymatic Methyl-seq (EM-seq) data on 41 matched urine–plasma pairs, which demonstrated that urine samples exhibited significantly higher tumor fractions and greater concordance with tissue methylation profiles than plasma. Based on this observation, we developed a urine-based bladder cancer detection framework using EM-seq. We profiled 143 urine samples (68 bladder cancer and 75 healthy controls) and 14 bladder cancer tissues. Methylation markers (113,052 regions) were identified by comparing cancer tissues (*n* = 14) with urine from healthy individuals (*n* = 14). Using XGBoost, possible features and their combinations were evaluated, with the combination of methylation and copy number variations (CNV) yielding the best performance as the final ensemble model. When evaluated on an independent test set, the model achieved 91.9% sensitivity at 80% specificity, with an area under the curve (AUC) of 0.932 for bladder cancer detection and 0.928 for non-muscle invasive bladder cancer (NMIBC) detection. Notably, the model successfully detected four of seven mutation-negative cases, demonstrating complementary value to mutation-based approaches.

## Simple Summary

Early detection of bladder cancer is challenging due to the limited release of tumor-derived DNA into body fluids. Using whole-genome methylation sequencing, we compared urine and plasma samples and found that urine captures stronger and more tissue-representative cancer signals. We developed a urine-based ensemble model integrating DNA methylation and copy number variations, achieving 91.9% sensitivity in detecting non-muscle invasive bladder cancer. Importantly, the model successfully identified mutation-negative cases, highlighting its complementary value to mutation-based approaches and its potential as a non-invasive tool for bladder cancer detection.

## 1. Introduction

Urothelial bladder cancer, predominantly non-muscle invasive bladder cancer (NMIBC), poses a distinct detection challenge in liquid biopsy [1]. NMIBC is characterized by limited tumor cell populations confined to superficial bladder epithelium, resulting in reduced shedding of tumor-derived DNA [2]. These characteristics fundamentally constrain early detection and necessitate approaches optimized for sensitivity in capturing low-abundance tumor markers.

Existing sequencing-based approaches to this challenge represent a trade-off between sequencing depth and genomic breadth. Conventional strategies typically optimize one information dimension within sequencing capacity limits, but sensitive NMIBC detection requires maximizing both dimensions, necessitating high-coverage whole-genome methylation sequencing approaches. Targeted approaches, including gene panels and methylation-specific PCR assays, maximize depth within selected regions but are restricted in genomic breadth [3,4,5]. Conversely, low-depth whole-genome approaches (shallow whole-genome sequencing [sWGS] or whole-genome bisulfite sequencing [sWGBS]) maximize breadth to profile global features such as tumor fraction, copy number variations, and global methylation, but lack sufficient per-locus depth to obtain region-specific information [6,7,8,9]. Detecting weak, distributed methylation signals in NMIBC requires both comprehensive genomic breadth and sufficient per-locus depth, a combination not achieved by existing approaches.

Beyond analytical strategy, sample selection critically determines the strength of detectable tumor-derived signals. Although NMIBC releases limited amounts of DNA, anatomical proximity suggests that urine may capture these signals more efficiently than plasma [10]. Tumor-derived DNA is shed directly into the urinary tract [11], whereas its detection in plasma depends on systemic circulation and is confounded by abundant leukocyte-derived background DNA, particularly from clonal hematopoiesis [12]. Prior studies have suggested urine’s advantages [7], but direct comparison using matched samples remains limited. Evaluation of matched urine–plasma pairs therefore provides a rationale for identifying the optimal biofluid for early bladder cancer detection.

In this study, we compared urine and plasma to develop an ensemble framework for early bladder cancer detection. Using EM-seq on 41 matched urine–plasma pairs, we demonstrated that urine exhibited higher tumor fractions and greater tissue methylation similarity than plasma, establishing urine as the optimal biofluid. We sequenced a total of 143 urine samples using high-coverage EM-seq, representing one of the largest whole-genome methylation sequencing datasets for urine-based bladder cancer detection. Analysis of 14 matched tissue–urine pairs validated high concordance in both copy number variations (CNV) and methylation profiles, with 113,052 methylation marker regions identified for model development. To maximize sensitivity, we integrated methylation patterns with multiple genomic features (copy number variations, global methylation, tumor fraction) within an ensemble framework. We achieved 91.9% sensitivity with 80% specificity through the combination of methylation and CNV, and detected mutation-negative cases from our previous study [13], suggesting potential for combined use in early bladder cancer detection.

## 2. Materials and Methods

### 2.1. Subjects

We prospectively collected clinical information, tissue, and urine samples from patients who underwent surgical treatment or intervention for malignant or benign urological disease. This study was approved by the Institutional Review Board of Ewha Woman’s University Seoul Hospital (IRB No. 2022-08-047). All patients provided informed consent for tissue banking and genetic testing. Urine and blood were collected prior to surgery. Tissue samples were obtained at the time of surgery. Five additional urine samples from healthy donors were kindly provided by Erger et al. [14]. Patients with malignancies other than bladder cancer were excluded. Mutation status of urine samples was previously determined using uAL100, a hybridization-based target capture panel covering 118 cancer-related genes [13].

This study included 68 patients with urothelial bladder cancer and 75 patients with benign disease. The patients with benign disease did not have any history of malignant disease and were considered healthy controls without cancer. For marker selection, we used tissue samples from 14 bladder cancer patients and urine samples from 14 healthy controls. The remaining urine samples were allocated for model development and validation: 31 bladder cancer and 41 healthy control urine samples for training/validation, and 37 bladder cancer and 20 healthy control urine samples for independent testing. The patients’ characteristics are summarized in Tables S1–S4.

### 2.2. EM-Seq Library Preparation

Genomic DNA from tissue samples was extracted using the DNeasy Blood & Tissue Kit (QIAGEN, Hilden, Germany) and sheared using a M220 Focused-Ultrasonicator (Covaris, Woburn, MA, USA). Urine samples (~50 mL) were collected via catheterization prior to surgery and stored in urine preservation tubes (Norgen Biotek, Thorold, ON, Canada) at room temperature. Within 1–2 days of collection, samples were centrifuged at 200× *g* for 10 min, followed by 3000× *g* for 20 min to remove cellular debris, and supernatants were stored at −70 °C until processing. Urinary cell-free DNA (cfDNA) was isolated from supernatants using a MagMAX^TM^ Cell-Free DNA Isolation Kit (Applied Biosystems, Thermo Fisher Scientific, Foster City, CA, USA), or a Quick-DNA^TM^ Urine Kit (Zymo Research, Irvine, CA, USA). Plasma samples (~10 mL whole blood) were collected in Cell-Free DNA BCT tubes (Streck, La Vista, NE, USA) prior to surgery and stored at room temperature. Within 1–2 days of collection, samples were centrifuged, and plasma was stored at −70 °C until cfDNA extraction using a MagMAX^TM^ Cell-Free DNA Isolation Kit (Applied Biosystems, Thermo Fisher Scientific, Foster City, CA, USA).

Whole-genome methylation sequencing libraries were prepared using the NEBNext Enzymatic Methyl-seq Kit (New England Biolabs, Ipswich, MA, USA) according to the manufacturer’s instructions. Library quality was assessed using TapeStation (Agilent, Santa Clara, CA, USA). Prepared libraries were sequenced on a NovaSeq 6000 platform (Illumina, San Diego, CA, USA) with 150 bp paired-end reads, achieving an average depth of 12.6×.

### 2.3. Sequencing Data Processing

Adapters from raw reads were trimmed with fastp (ver. 0.23.4) [15]. Alignment was done with BitMapperBS (ver. 1.0.2.3) to the hg19 reference genome [16]. Sorting and indexing were performed with Samtools (ver. 1.21) [17]. Duplicate reads were marked with GATK MarkDuplicates (ver. 4.5.0.0) [18]. BamUtil clipOverlap (ver. 1.0.15) was used to clip overlapping paired-end reads [19]. CpG and CHH contexts were extracted with MethylDackel (ver. 0.6.1) [20]. Cytosine conversion efficiency was calculated within the CHH context by dividing the total number of C-to-T converted bases by the total number of sequenced cytosines. Samples with cytosine conversion efficiency below 97% were excluded from the analysis.

### 2.4. Methylation Call and Marker Selection

For the methylation call, we divided the hg19 reference genome into 300 bp windows. Windows overlapping ENCODE blacklist regions were removed prior to analysis [21]. Average methylation fraction (AMF) was calculated for CpG sites within each window by dividing the total number of cytosine reads by the total number of cytosine and thymine reads. For each sample, windows covering CpG sites with fewer than 30 reads were removed. Global methylation was defined as the mean AMF across all windows for each sample. The global methylation score was inverted (1-global methylation) prior to incorporation into the ensemble model, as global hypomethylation is observed in cancer.

To select methylation markers, 14 bladder cancer tissue samples and 14 urine samples from healthy individuals were used. Regions supported by a minimum of 12 healthy samples and 4 cancer samples were retained. Welch’s t-test was performed and *p* values were corrected using the Benjamini–Hochberg method. Regions with FDR < 1 × 10^−5^ and AMF difference > 0.25 were selected, yielding a total of 113,052 methylation marker regions.

### 2.5. Assessment of Tissue–Urine Concordance in Methylation Markers

Tissue–urine concordance was assessed using 14 matched tissue–urine pairs from bladder cancer patients. For each genomic region, delta AMF values were calculated by subtracting the mean AMF of 14 urine samples from healthy individuals as baseline. Tissue–urine concordance was defined as the ratio of urine delta AMF to tissue delta AMF for each matched pair, as follows:

$$Tissue-Urine\;Concordance=\;\frac{{AMF}_{Urine}-{AMF}_{Healthy}}{{AMF}_{Tissue}-{AMF}_{Healthy}}$$

For each region, the median concordance value across all 14 pairs was calculated. Positive concordance values indicate directional agreement in methylation changes between tissue and urine samples.

### 2.6. CNV Call

Copy number variations were analyzed using ichorCNA with 1 Mbp genomic windows, using a panel of normals from healthy donors for baseline correction [22]. Log2 ratios of normalized read counts relative to the normal panel were calculated for each window and used as CNV profiles. Estimated tumor fractions from ichorCNA were used as features in the ensemble model.

### 2.7. Model Training for Methylation and CNV

XGBoost models were trained for methylation and CNV using the xgboost R package [23]. Model evaluation was performed using 10 repeated 3-fold cross-validation. Missing values in each fold were imputed with the median values from healthy controls within the training set. The methylation model used AMF values from 113,052 marker regions as features, while the CNV model used log2 ratios from all genomic windows. Hyperparameters were optimized independently for each model, resulting in the following settings: methylation model (max_depth = 3, eta = 0.01, nrounds = 200, subsample = 0.7, colsample_bytree = 0.6, min_child_weight = 5) and CNV model (max_depth = 3, eta = 0.1, nrounds = 200, subsample = 0.7, colsample_bytree = 0.6, min_child_weight = 5).

### 2.8. Ensemble Model Construction

Following cross-validation, the methylation and CNV XGBoost models were retrained on the combined training and validation sets to generate final predictions. An ensemble model was constructed by integrating four features: methylation model scores, CNV model scores, global methylation, and tumor fraction. The ensemble score was calculated as:

Ensemble score=∑(w ×S)

where S represents the score from each feature and w represents the weight assigned to each feature. Equal weights were assigned to each selected feature, with all weights summing to 1. All possible combinations of available features, including global methylation and tumor fraction, were evaluated and compared by AUC, and the combination of methylation and CNV scores yielded the best performance. The resulting ensemble scores were applied to the independent test set, which was restricted to samples with confirmed mutation status to enable direct comparison with mutation-based approaches.

### 2.9. Annotation and Gene Ontology Enrichment Analysis

Genes were annotated using R package annotatr (ver. 1.14.0) and GenomicRanges (ver. 1.42.0) [24,25]. Unique genes overlapping with methylation marker regions were subjected to gene ontology enrichment analysis using Enrichr [26].

### 2.10. Statistical Analysis

Wilcoxon rank-sum test and Wilcoxon signed-rank test were used for comparing groups. For tests involving multiple comparisons, *p* values were corrected by the Benjamini–Hochberg method. Correlations were assessed using Spearman and Pearson methods.

## 3. Results

### 3.1. Design and Rationale for Urine-Based Cancer Detection Model

**Figure 1:**
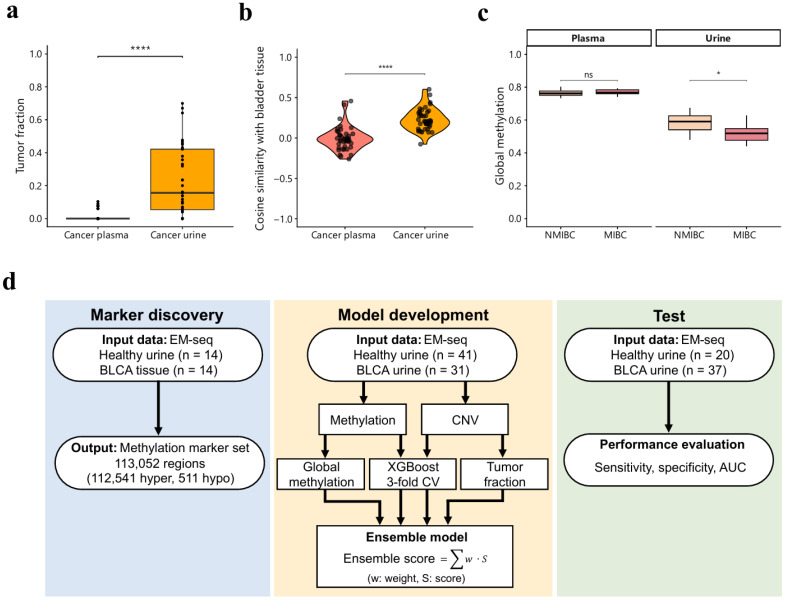
Workflow and background for urine-based model construction. (**a**) Comparison of tumor fraction between matched plasma and urine samples from 41 cancer patients is shown (*n* = 41 pairs). Urine samples exhibited significantly higher tumor fraction compared to plasma samples. Wilcoxon signed-rank test; **** *p* < 0.0001. (**b**) Cosine similarity to bladder tissue methylation patterns was calculated for each urine or plasma sample (*n* = 41 matched pairs) against the region-wise average methylation profile of bladder tissues (*n* = 14). Wilcoxon signed-rank test; **** *p* < 0.0001. (**c**) Global methylation levels in matched plasma and urine samples across disease groups are presented (NMIBC, *n* = 33; MIBC, *n* = 8). Wilcoxon rank-sum test; ns *p* ≥ 0.05, * *p* < 0.05. (**d**) Schematic of the workflow for urine-based bladder cancer detection model development.

We first examined tumor fractions in matched plasma–urine pairs to assess the relative strength of tumor-derived signals in each biofluid. Urine samples from bladder cancer patients demonstrated significantly higher tumor fractions compared to their matched plasma samples (Figure 1a). Beyond signal strength, we investigated whether urine samples better preserve bladder cancer-specific methylation patterns. Using the region-wise average methylation profile from 14 bladder cancer tissue samples as a reference, we calculated cosine similarity of matched urine and plasma samples. Urine samples showed significantly higher similarity to tissue methylation patterns than their plasma counterparts (Figure 1b), demonstrating that urine more effectively captures bladder cancer-associated methylation information. This enhanced signal strength was also evident in global methylation profiles, where urine samples exhibited stage-dependent decreases in global methylation, while matched plasma samples remained stable throughout cancer progression (Figure 1c). Together, these findings indicate that tumor-derived signals are substantially stronger in urine than in plasma. These observations provided strong rationale for developing a urine-based cancer detection model.

Based on these findings, we established a comprehensive framework for urine-based bladder cancer detection following the workflow illustrated in Figure 1d. The framework consists of three sequential phases: marker discovery, model development, and independent validation. We aimed to develop an ensemble model integrating multiple genomic features to maximize detection sensitivity. Detailed sample information is provided in Tables S1–S4.

### 3.2. Tissue-Derived Signals Present in Urine CNV

**Figure 2:**
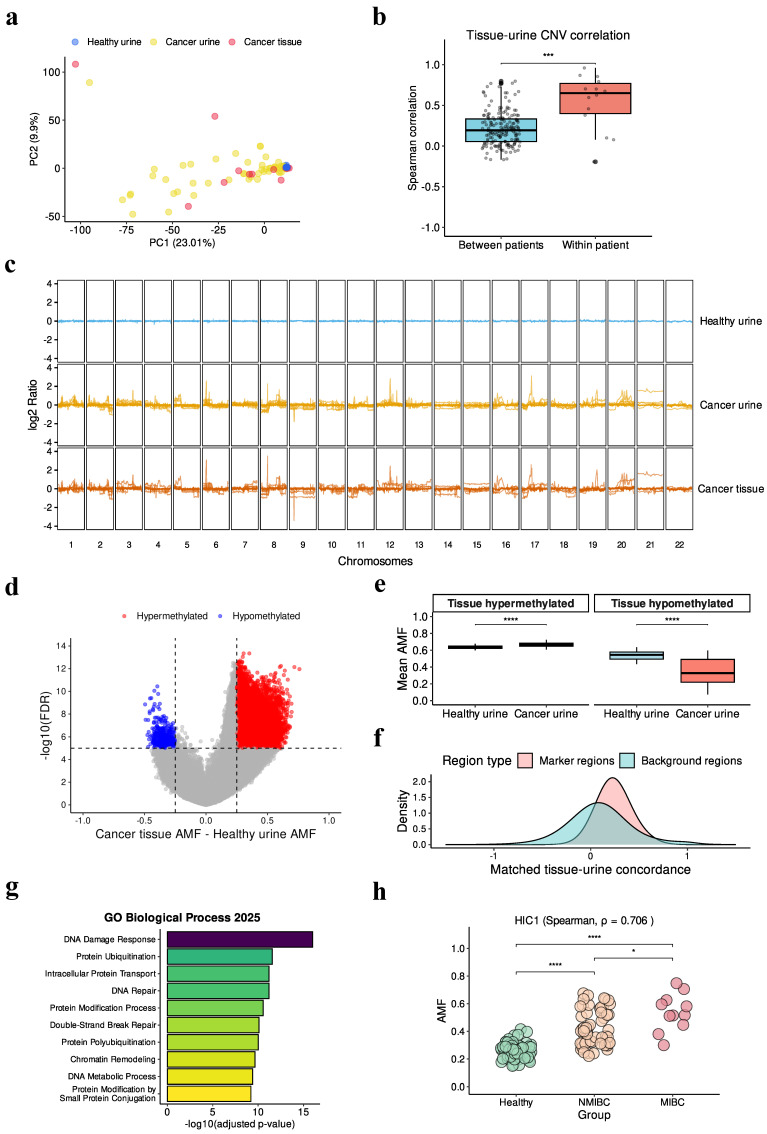
Urine samples show concordant signals with bladder cancer tissues. (**a**) Principal component analysis of CNV log2 ratio profiles (1 Mbp bins) across sample types (healthy, *n* = 75; cancer, *n* = 68; tissue, *n* = 14). (**b**) Spearman correlation of genome-wide CNV log2 ratio profiles between tissue and urine samples. Each dot represents the correlation coefficient for one tissue–urine pair. “Within patient” refers to tissue–urine pairs from the same donor (*n* = 14), and “Between patient” refers to tissue–urine pairs from different donors (*n* = 182). Wilcoxon rank-sum test; *** *p* < 0.001. (**c**) Genome-wide CNV log2 ratio profiles (1 Mbp bins) across chromosomes for representative samples. (**d**) Identification of differentially methylated regions between bladder cancer tissues (*n* = 14) and urine samples from age- and gender-matched healthy individuals (*n* = 14). Welch’s t-test identified 113,052 differentially methylated regions, comprising 112,541 hypermethylated (red) and 511 hypomethylated regions (blue). (**e**) Validation of tissue-derived methylation markers in urine samples. Mean AMF values were calculated across marker regions for urine samples from healthy individuals (*n* = 61) and bladder cancer patients (*n* = 68). Wilcoxon rank-sum test; **** *p* < 0.0001. (**f**) Region-level tissue–urine concordance analysis using matched tissue–urine pairs (*n* = 14). (**g**) Gene ontology enrichment analysis of genes overlapping with the 113,052 bladder cancer methylation markers (11,761 unique genes). (**h**) Methylation levels (AMF) of *HIC1*, a DNA damage response gene, across disease stages (healthy, *n* = 61; NMIBC, *n* = 56; MIBC, *n* = 11), with positive correlation (Spearman *ρ* = 0.706, *p* < 0.0001). Wilcoxon rank-sum test; * *p* < 0.05, **** *p* < 0.0001.

Principal component analysis (PCA) of CNV profiles revealed distinct clustering patterns across sample types (Figure 2a). Bladder cancer tissue samples clustered closely with urine samples from cancer patients, while urine samples from healthy individuals formed a distinct cluster. These clustering patterns were independently confirmed by UMAP analysis (Figure S1), demonstrating strong CNV similarity between cancer tissues and patient-derived urine samples. Notably, no distinct clustering by DNA extraction kit or sequencing batch was observed among urine samples from healthy individuals (Figure S2), suggesting that technical variability does not substantially influence CNV profiles in our dataset.

We hypothesized that urine samples would be enriched for patient-specific genomic alterations. To test this, we examined whether tissue–urine pairs from the same donor showed higher correlation than cross-donor comparisons among the 14 cancer patients (Figure 2b). Matched tissue–urine pairs exhibited significantly higher correlations compared to unmatched pairs, confirming the presence of patient-specific signals. This patient-specificity was further evident in the CNV heatmap (Figure 2c), which revealed distinct patient-specific CNV events in cancer patient urines that were absent in healthy donor samples.

### 3.3. Tissue-Derived Signals Present in Urine Methylation

To address the inherent variability in genome-wide methylation data, we selected marker regions that exhibited consistent hypermethylation or hypomethylation patterns in bladder cancer tissues relative to healthy urine samples (Figure 2d). Within these marker regions, the AMF in urine samples from cancer patients was concordant with tissue methylation patterns (Figure 2e). Specifically, urine from cancer patients showed hypermethylation in tissue-hypermethylated regions and hypomethylation in tissue-hypomethylated regions.

To validate the effectiveness of our marker selection strategy, we assessed tissue–urine concordance within the 14 matched pairs (Figure 2f). We calculated regional tissue–urine concordance (see Materials and Methods) and found that 93.6% of marker regions showed positive concordance, compared to only 61.7% of background regions. This enrichment demonstrates that our marker selection approach successfully identified regions with high tissue–urine concordance.

Gene ontology enrichment analysis of the methylation marker regions revealed significant associations with DNA damage response and DNA repair pathways (Figure 2g), consistent with findings from previous studies [27,28]. We also observed progressive stage-dependent hypermethylation of representative DNA damage response genes (Figure 2h and Figure S3), suggesting biological relevance of our marker set in bladder cancer progression.

### 3.4. Evaluation of Ensemble Model Performance

**Figure 3:**
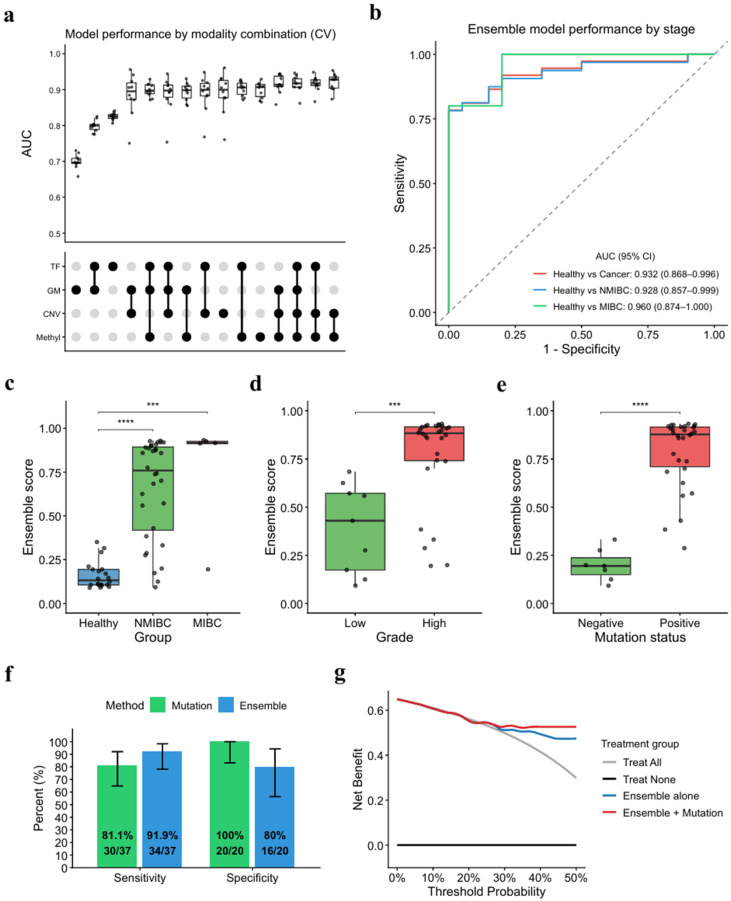
Performance of urine-based ensemble model in bladder cancer detection. (**a**) Model performance comparison across different modalities during cross-validation (3-fold, 10 repeats). Each point represents the mean AUC from three folds within a single repeat (*n* = 10). TF, tumor fraction; GM, global methylation score; CNV, CNV model score; Methyl, methylation model score. (**b**) Receiver operating characteristic (ROC) curves for the ensemble model on the test set (healthy, *n* = 20; NMIBC, *n* = 32; MIBC, *n* = 5). (**c**–**e**) Ensemble scores across disease progression, tumor grade, and mutation status in the test set. (**c**) Scores across healthy (*n* = 20), NMIBC (*n* = 32), and MIBC (*n* = 5) groups. (**d**) Scores by tumor grade in cancer patients (high-grade, *n* = 28; low-grade, *n* = 9). (**e**) Scores by mutation status in cancer patients (mutation positive, *n* = 30; mutation negative, *n* = 7). Wilcoxon rank-sum test; *** *p* < 0.001, **** *p* < 0.0001. (**f**) Comparison of sensitivity and specificity between mutation and ensemble model prediction. (**g**) Decision curve analysis of the ensemble model and mutation combination strategy in the test set. Here, the combined model called cancer when either the mutation or ensemble model was positive.

To identify the optimal feature combination, we evaluated all 15 possible combinations of four candidate features, including methylation model scores, CNV model scores, global methylation, and tumor fraction, based on cross-validation AUC (Figure 3a). The combination of methylation and CNV scores yielded the highest AUC (median = 0.928) and was selected as the final ensemble model. Although some features showed relatively high pairwise correlations (Figure S4), performance varied across combinations, suggesting that methylation and CNV scores capture complementary aspects of bladder cancer signals. The ensemble model was then evaluated on the independent test set, resulting in an AUC of 0.932 for bladder cancer detection (Figure 3b), with similarly strong performance for NMIBC detection (AUC = 0.928).

Ensemble model scores showed significant associations with key clinical and molecular features. Scores increased significantly with advancing tumor stage (Figure 3c), higher histologic grade (Figure 3d), and the presence of somatic mutations (Figure 3e). Notably, ensemble scores exhibited positive correlation with the number of detected mutations (Figure S5), suggesting that our multi-feature model effectively captures genomic alterations associated with bladder cancer progression. Analysis of potential confounders revealed that age and gender did not significantly influence ensemble model scores (Figures S6 and S7), indicating that the model was not trained on demographic biases in the dataset.

When comparing our ensemble model performance to mutation-based detection, we found that our model achieved higher sensitivity (91.9% vs. 81.1%), though with lower specificity (80% vs. 100%) (Figure 3f and Figure S8). Notably, the model successfully identified four of the seven mutation-negative samples. These results suggest complementary roles for methylation-based ensemble modeling and mutation detection. When combined, the integrated approach offered additional diagnostic value, demonstrating higher net benefit across a broad range of threshold probabilities in decision curve analysis (Figure 3g and Figure S9). Together, these findings indicate that integrating the two tests may offer a clinically practical strategy, particularly in high-risk populations where maximizing detection yield is paramount.

## 4. Discussion

In this study, we developed a urine-based ensemble model integrating methylation, CNV, global methylation, and tumor fraction for bladder cancer detection. To our knowledge, this represents the first study to apply EM-seq to urine samples at this scale for bladder cancer detection. Our matched urine–plasma analysis demonstrated that urine samples contained significantly higher tumor fractions and exhibited greater similarity to bladder tissue methylation patterns compared to plasma samples (Figure 1a–c). Analysis of matched tissue–urine pairs revealed high concordance in both CNV and methylation profiles (Figure 2). These findings validate urine as a preferred biofluid for bladder cancer liquid biopsy, demonstrating that urine DNA not only shows enriched tumor-derived signals but also preserves tissue-specific genomic and epigenomic patterns. Comparison with mutation-based detection from our previous study [13] reveals complementary strengths between the two approaches. While mutation detection achieved 81.1% sensitivity with 100% specificity, our ensemble model demonstrated improved sensitivity of 91.9%, albeit with lower specificity of 80%. Importantly, our ensemble model successfully detected four of seven mutation-negative samples, underscoring the benefit of multi-modal detection strategies.

The clinical implications of this performance trade-off merit further consideration. Patients undergoing postoperative surveillance or those classified as high-risk for bladder cancer recurrence represent a population where molecular signals of malignancy are more likely to be present, and where missing a diagnosis carries significant clinical consequences. In such settings, maximizing sensitivity is paramount, and our model’s 91.9% sensitivity offers a meaningful advantage over mutation-based detection for capturing these at-risk patients. As demonstrated by our decision curve analysis, incorporating mutation testing into the ensemble model further enhanced net clinical benefit across a wide range of threshold probabilities (Figure 3g and Figure S9). Although the 80% specificity introduces false positives, the primary value of urine-based liquid biopsy in this targeted population lies in reducing the overall burden of cystoscopy by triaging patients, rather than eliminating it entirely.

Despite these findings, several important limitations should be acknowledged. First, while our cohort of 143 urine samples is substantial for high-coverage EM-seq studies, it remains limited for robust clinical validation of machine learning models. Expanding to several hundred samples faces practical barriers including prohibitive sequencing costs and lengthy turnaround times for both sequencing and data processing. Such limited cohort size also constrained balanced sample distribution, particularly when restricted to mutation-confirmed samples for comparative analysis. This represents a fundamental constraint between data richness and cohort size. Second, we evaluated only age and gender as potential confounders, while other bladder cancer-associated factors such as smoking history [29], urinary tract infections [30], and medication use [31] were not considered. Comprehensive confounder adjustment would require sample sizes of several hundred to maintain statistical power, again highlighting the sample size constraint. Third, our ensemble approach relied on XGBoost for generating methylation and CNV component scores. Exploring alternative algorithms such as glmnet or random forest could potentially improve model performance through hyperparameter optimization. Moreover, deriving multiple algorithm-based scores for each trained feature (methylation and CNV) rather than a single model score per feature might better capture the complementary strengths of different machine learning approaches, potentially enhancing overall predictive performance [32,33].

A key future direction is integration with mutation detection data to develop a combined testing strategy that leverages the complementary strengths of both approaches. Integrated models that combine methylation, CNV, and mutation features in a unified framework warrant further investigation. As a single-center study, our findings may be subject to population-specific biases, and external validation with independent cohorts is essential to assess generalizability and clinical applicability across diverse populations. In addition, given the strong tumor-derived signals in urine, the methylation data generated in this study could serve as a resource for further elucidating the functional significance of epigenetic changes in bladder cancer development. To validate the functional impact of such epigenetic alterations, patient-derived organoids (PDOs) could serve as an ideal experimental platform. Previous studies have established PDOs as a robust model that recapitulates the genomic landscape and tumor evolution of the primary bladder cancer [34,35]. Furthermore, the ability to profile diverse epigenetic profiles in response to drug treatment represents a key advantage of this platform, potentially bringing us closer to multi-omics-guided therapeutic selection for individual patients.

## 5. Conclusions

We developed a whole-genome methylation sequencing ensemble model for urine-based bladder cancer detection, achieving 91.9% sensitivity with 80% specificity and demonstrating the superiority of urine over plasma through direct matched-pair comparisons. Our findings confirm high concordance between urine and tissue molecular profiles in both CNV and methylation contexts, validating the basis for urine as a preferred biofluid for bladder cancer liquid biopsy. The ensemble model showed improved sensitivity compared to mutation-based detection, suggesting potential for integrated non-invasive diagnostic approaches. Despite limitations in sample size inherent to high-coverage sequencing studies, our results establish a foundation for urine-based multi-modal detection and hold promise for bladder cancer screening and surveillance in high-risk populations.

## Data Availability

The data and source codes used in this study are available upon request to the corresponding author, Duhee Bang (duheebang@yonsei.ac.kr).

## Supplementary Materials

The following supporting information can be downloaded at: https://www.mdpi.com/article/10.3390/cancers18050767/s1, Figure S1: CNV profile presentation by UMAP; Figure S2: UMAP projection of CNV profiles by extraction kit and batch; Figure S3: Progressive hypermethylation of *SPIDR* gene in bladder cancer; Figure S4: Pairwise Spearman correlations between ensemble candidate features; Figure S5: Correlation between ensemble score and the number of mutations detected; Figure S6: Ensemble score distribution by age and gender in the test set; Figure S7: Heatmap of top 5000 variable methylation regions in test set samples; Figure S8: Sample-level comparison of clinical diagnosis, mutation status, and ensemble predictions in the test set; Figure S9: Decision curve analysis of mutation testing and combined ensemble-mutation strategies in the test set; Table S1: Characteristics of samples used for marker selection; Table S2: Characteristics of urine samples for model training, validation, and testing; Table S3: Characteristics of plasma samples in this study; Table S4: Clinical diagnoses of non-bladder cancer controls.
