# Supplementary material for: Urine-Based cfDNA Ensemble Modeling for Early Detection of Bladder Cancer Using Whole-Genome Methylation Sequencing
Source: Cancers (Basel). 2026 Feb 27;18(5):767. doi: 10.3390/cancers18050767 (PMC12984421; doi:10.3390/cancers18050767)
Supplement: Supplementary file 1 [file cancers-18-00767-s001.zip › cancers-4147041-supplementary.pdf]

Supplementary Material

1 Supplementary Figures

Figure S1. CNV profile presentation by UMAP. UMAP projection of CNV profiles for urine from healthy individuals (blue; n = 75), urine from bladder cancer patients (yellow; n = 68), and bladder cancer tissues (red; n = 14).

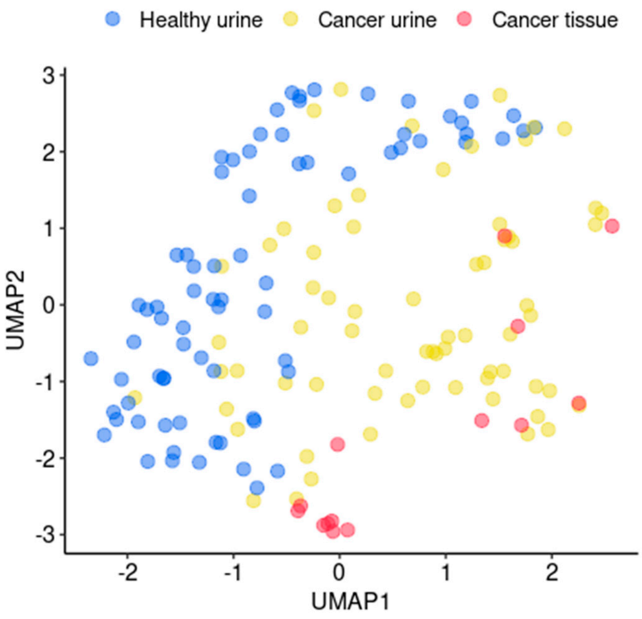

Figure S2. UMAP projection of CNV profiles by extraction kit and batch. CNV profiles from urine samples of healthy individuals (n = 75) were presented with UMAP by (**a**) DNA extraction kit and (**b**) batch.

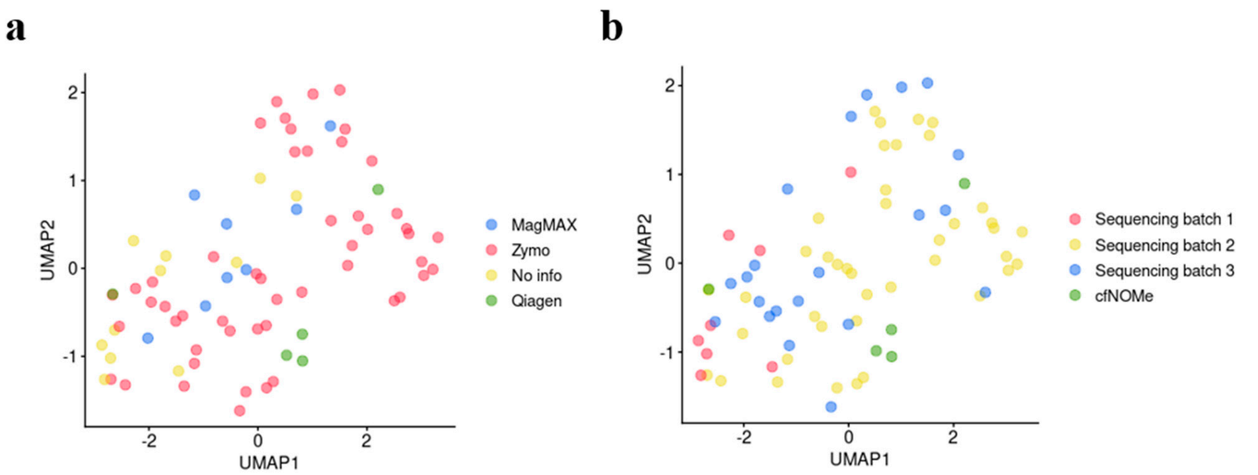

Figure S3. Progressive hypermethylation of *SPIDR* gene in bladder cancer. Average methylation fraction (AMF) for *SPIDR* gene region across disease progression groups in urine samples (healthy, $n = 61$; NMIBC, $n = 56$; MIBC, $n = 11$). Increase in methylation was observed with disease progression (Spearman $\rho = 0.554$, $p < 0.0001$). Wilcoxon rank-sum test; $^{**}p < 0.01$, $^{****}p < 0.0001$.

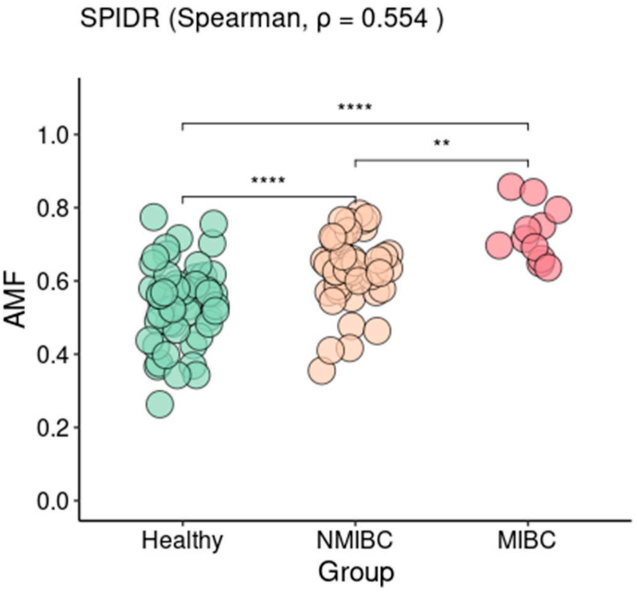

Figure S4. Pairwise Spearman correlations between ensemble candidate features. Spearman correlation coefficients averaged across cross-validation repeats are shown ($n = 10$). TF, tumor fraction; GM, global methylation score; CNV, CNV model score; Methyl, methylation model score.

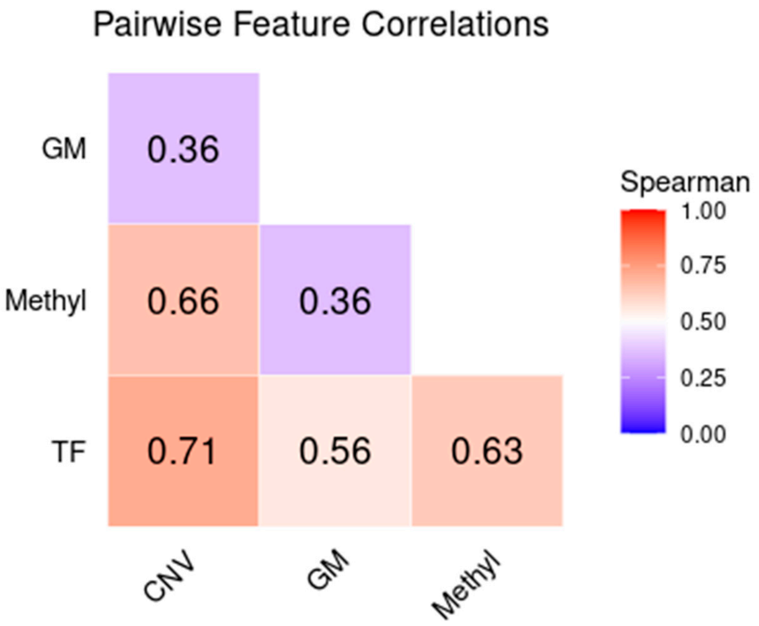

Figure S5. Correlation between ensemble score and the number of mutations detected. Correlation between number of mutations detected and ensemble score in urine samples (healthy, n = 20; cancer, n = 37). Samples include mutation-negative (n = 27) and mutation-positive (n = 30) cases (Pearson correlation r = 0.69, p < 0.0001).

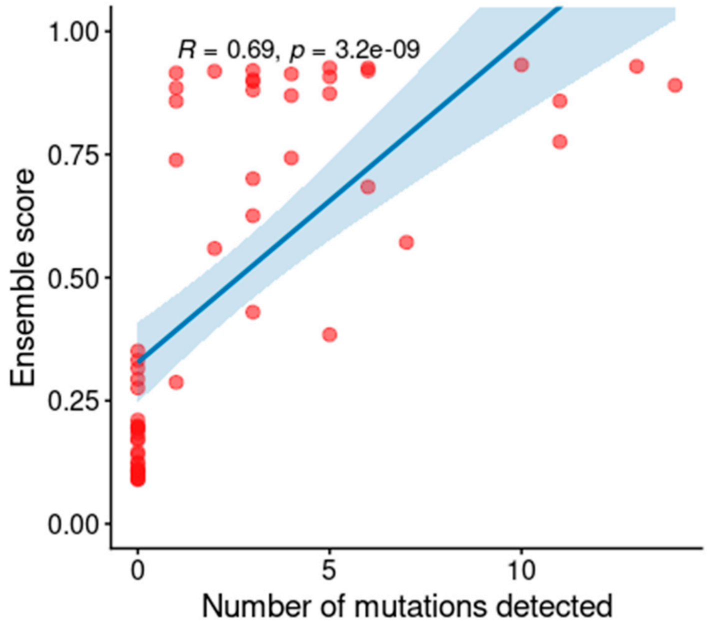

Figure S6. Ensemble score distribution by age and gender in the test set. No significant differences were observed within healthy or cancer groups for either age or gender. (**a**) Ensemble scores stratified by age groups within healthy and cancer cohorts (healthy <60, $n = 5$; healthy 60+, $n = 15$; cancer <60, $n = 9$; cancer 60+, $n = 28$). (**b**) Ensemble scores stratified by gender within healthy and cancer cohorts (healthy female, $n = 1$; healthy male, $n = 19$; cancer female, $n = 12$; cancer male, $n = 25$). Wilcoxon rank-sum test; ns $p \geq 0.05$.

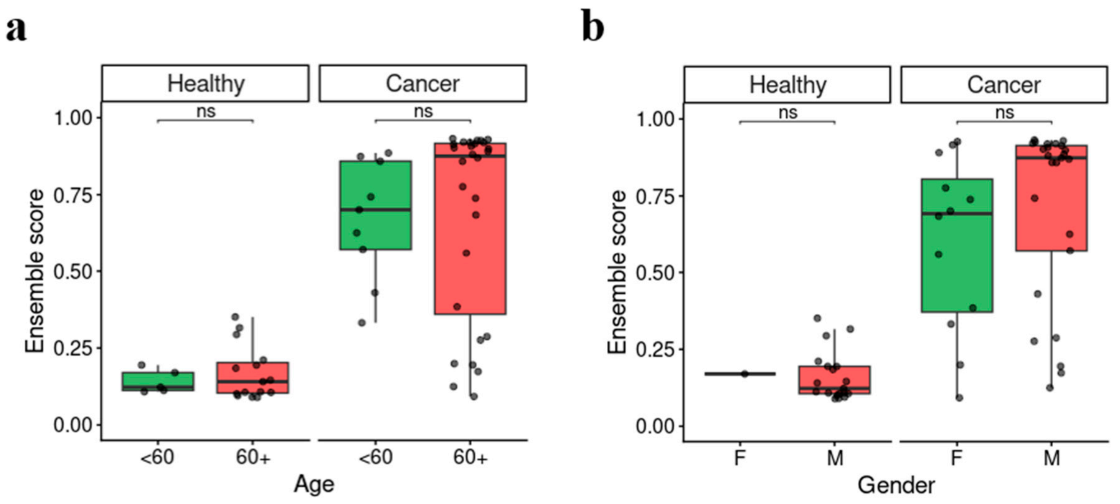

Figure S7. Heatmap of top 5,000 variable methylation regions in test set samples. Heatmap of test set samples across the 5,000 most variable methylation regions, which were selected from bladder cancer tissues (*n* = 14) based on the highest standard deviation with at least 10 supporting samples per region. Hierarchical clustering was performed on both samples and regions. Sample annotations include ensemble prediction, mutation status, disease status, stage, grade, gender, and age. Color scale represents AMF from 0 (blue) to 1 (red).

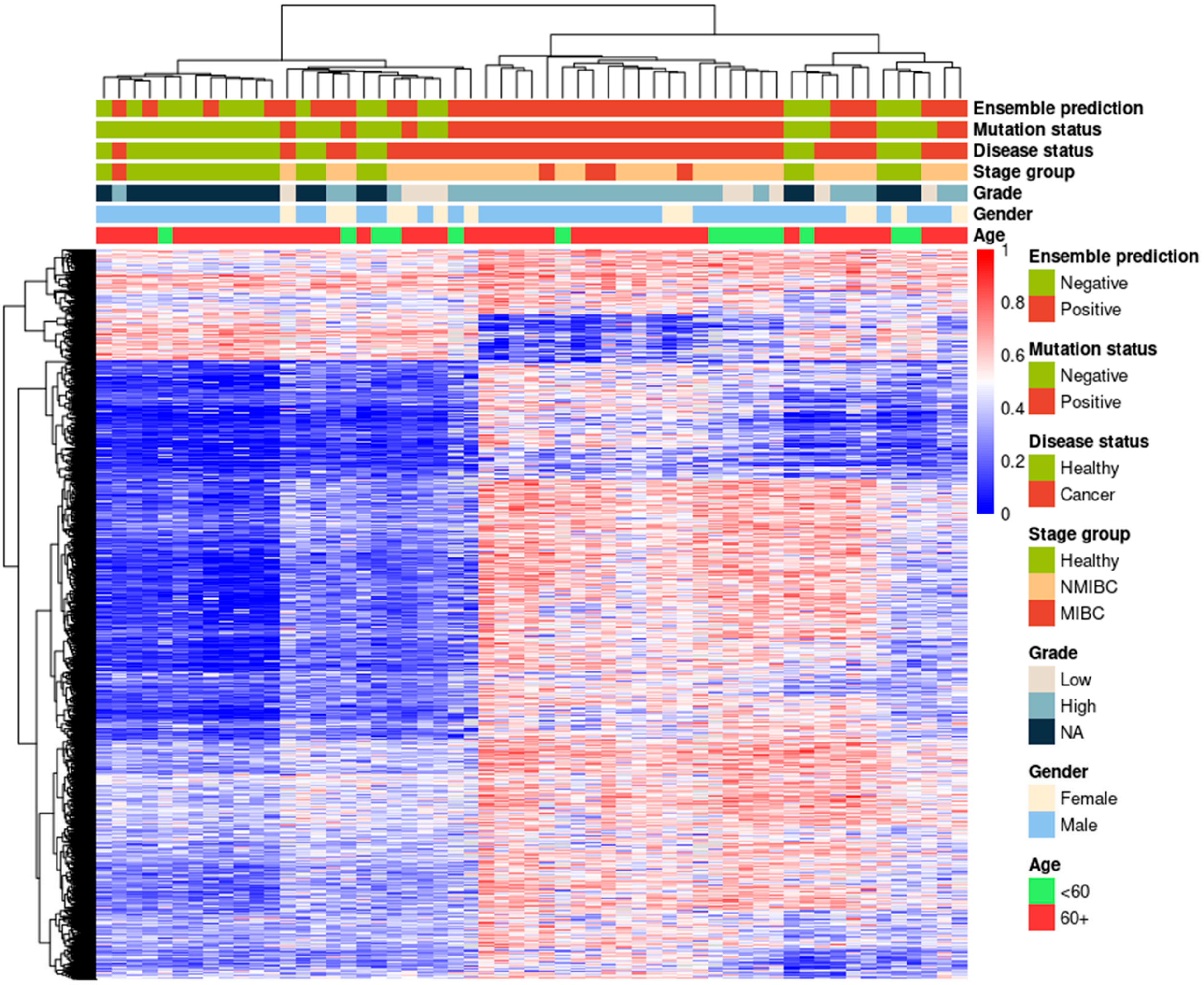

Figure S8. Sample-level comparison of clinical diagnosis, mutation status, and ensemble predictions in the test set. Each column represents an individual sample, with red indicating positive and green indicating negative for each classification method (healthy, n = 20; cancer, n = 37).

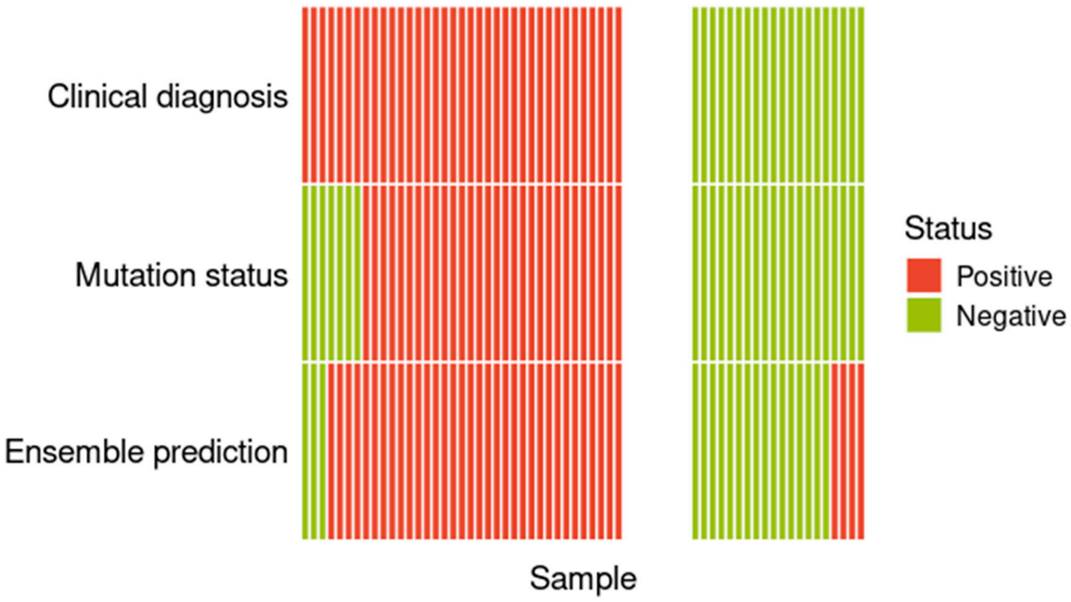

Figure S9. Decision curve analysis of mutation testing and combined ensemble-mutation strategies in the test set. (**a**) Decision curve analysis of mutation testing alone. **(b)** Decision curve analysis of combined ensemble-mutation strategies using OR (cancer if either test is positive) and AND (cancer only if both tests are positive) approaches.

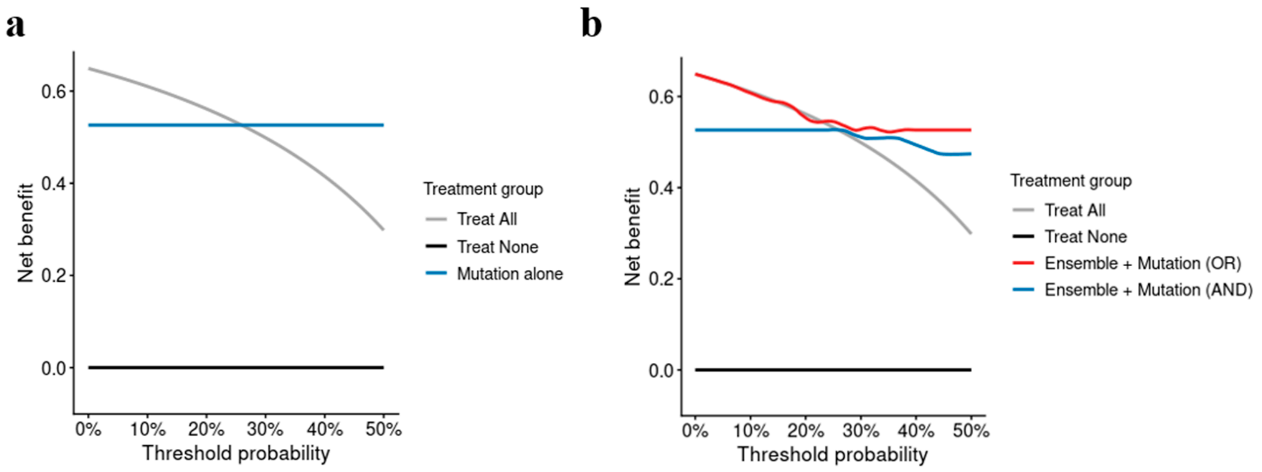

2 Supplementary Tables

Table S1. Characteristics of samples used for marker selection

	Non-bladder cancer	Bladder cancer
Sample type	Urine	Tissue
Total	14	14
Gender (male / female)	12 / 2	12 / 2
Median age	67	68.5
NMIBC (Stage 0 / I)	-	10 (6 / 4)
MIBC (Stage II / III / IV)	-	4 (3 / 0 / 1)
Low grade	-	3
High grade	-	11

Table S2. Characteristics of urine samples for model training, validation, and testing

	Train/validation	Test
Sample type	Urine	Urine
Total	72	57
Non-bladder cancer	41	20
Bladder cancer	31	37
Gender (male / female)	60 / 12	44 / 13
Median age	68	66
NMIBC (Stage 0 / I)	24 (15 / 9)	32 (16 / 16)
MIBC (Stage II / III / IV)	6 (5 / 0 / 1)	5 (3 / 1 / 1)
Low grade	9	9
High grade	21	28
Mutation negative	-	27
Mutation positive	-	30
Average tumor fraction (Non-bladder cancer / Bladder cancer)	0.10 (0.03 / 0.21)	0.14 (0.02 / 0.21)

Table S3. Characteristics of plasma samples in this study

	Bladder cancer
Total	41
Gender (male / female)	30 / 11
Median age	67
NMIBC (Stage 0 / I)	33 (18 / 15)
MIBC (Stage II / III / IV)	8 (6 / 0 / 2)
Low grade	8
High grade	33
Matched tissue	14
Matched urine	41

Table S4. Clinical diagnoses of non-bladder cancer controls

Diagnosis	Number of patients (%)
Benign prostatic hyperplasia (BPH)	60 (80%)
Urolithiasis	6 (8%)
Benign renal tumor	2 (2.7%)
Ureteropelvic Junction Obstruction (UPJO)	1 (1.3%)
Ureter polyp	1 (1.3%)
Acute kidney graft dysfunction	1 (1.3%)
Acute kidney injury (AKI)	1 (1.3%)
Unknown	3 (4%)
Total	75 (100%)
